# Binding behavior of spike protein and receptor binding domain of the SARS-CoV-2 virus at different environmental conditions

**DOI:** 10.1038/s41598-021-04673-y

**Published:** 2022-01-17

**Authors:** Meiyi Zhang, Haoqi Wang, Emma R. Foster, Zivko L. Nikolov, Sandun D. Fernando, Maria D. King

**Affiliations:** Department of Biological and Agricultural Engineering, Texas A&M University, 2117 TAMU, College Station, TX 77843 USA

**Keywords:** Biochemistry, Proteins

## Abstract

A novel coronavirus, severe acute respiratory syndrome coronavirus 2 (SARS-CoV-2) was identified as the cause of the COVID-19 pandemic that originated in China in December 2019. Although extensive research has been performed on SARS-CoV-2, the binding behavior of spike (S) protein and receptor binding domain (RBD) of SARS-CoV-2 at different environmental conditions have yet to be studied. The objective of this study is to investigate the effect of temperature, fatty acids, ions, and protein concentration on the binding behavior and rates of association and dissociation between the S protein and RBD of SARS-CoV-2 and the hydrophobic aminopropylsilane (APS) biosensors using biolayer interferometry (BLI) validated with molecular dynamics simulation. Our results suggest three conditions—high ionic concentration, presence of hydrophobic fatty acids, and low temperature—favor the attachment of S protein and RBD to hydrophobic surfaces. Increasing the temperature within an hour from 0 to 25 °C results in S protein detachment, suggesting that freezing can cause structural changes in the S protein, affecting its binding kinetics at higher temperature. At all the conditions, RBD exhibits lower dissociation capabilities than the full-length S trimer protein, indicating that the separated RBD formed stronger attachment to hydrophobic surfaces compared to when it was included in the S protein.

## Introduction

In December 2019, a cluster of cases of pneumonia with unknown cause was found in Wuhan, China^1^. Later the cause was identified as a novel, severe acute respiratory syndrome coronavirus 2 (SARS-CoV-2), the seventh known coronavirus that infects humans that is responsible for the global pandemic coronavirus disease 2019 (COVID-19)^2,3^. As of May 2021, more than 150 million cases related to SARS-CoV-2 were confirmed worldwide, resulting in about 3 million deaths^4^. Several studies have investigated the clusters of infection of workers in the unique environment of meat processing facilities around the world from different aspects. For example, Herstein et al. examined the characteristics of confirmed SARS-CoV-2 cases among meat processing workers in Nebraska and concluded that the risk of adverse outcomes was disproportionately high among ethnic and racial minority groups as well as among men^5^. Mitigating the spread of SARS-CoV-2 in industrial settings such as meat processing facilities has been especially challenging as a study sequenced the whole viral genomes of SARS-CoV-2 infections from a healthcare system in Iowa and discovered that a single infected individual who worked at a meatpacking plant led to unrestrained spread within the meat facility and consequently to individuals in 13 surrounding cities^6^. Pokora et al. analyzed the work environment and protective measures in twenty-two meat and poultry plants with about 20,000 workers in Germany and revealed that the chance of SARS-CoV-2 infection was higher in the deboning and meat cutting area as well as an association with the ventilation rate per employee, room temperature, climate conditions, and outdoor air flow, which all had the potential to promote the spread of SARS-CoV-2^7^. An investigation of a SARS-CoV-2 outbreak in a meat processing plant in Germany reported that environmental conditions such as temperature, air exchange rates, air circulation, distance between workers, and the type of work are all important factors in transmitting SARS-CoV-2 aerosols, explaining why meat processing facilities have become hotspots for the virus outbreak worldwide^8^. The Food and Agriculture Organization of the United Nations recommended that all rooms in which meat is processed should maintain a temperature of about 12 °C, a standard followed by most meat processing plants^9^. Although low temperature was identified as one of the factors that promote aerosol transmission of SARS-CoV-2, the effect of different temperatures on the attachment of SARS-CoV-2 to surfaces remains largely unknown. Low temperature and high humidity are typical in critical infrastructures such as food processing facilities. Although studies have been conducted on the role of lipid synthesis and metabolism in SARS-CoV-2 replication and inhibitors have been developed regarding the antiviral efficacy of lipids, the effect of lipids on SARS-CoV-2 binding remained unknown^10–12^. The air properties together with high concentration of fat particles in the air, usually generated from meat cutting and commonly present in meat processing plants, may enhance attachment of SARS-CoV-2 to surfaces.

The enveloped, single stranded RNA virus SARS-CoV-2 contains four types of structural proteins—spike (S) protein, envelope (E) protein, membrane (M) protein, and nucleocapsid (N) protein^13^. The trimeric S protein is the major glycoprotein responsible for receptor binding and membrane fusion, which mediates virus entry into the target cells^14^. The S1 subunit of S protein recognizes and binds to host cells receptors which initiates the S2 subunit to fuse the membrane of the virus and host^15^. The S protein contains the receptor-binding domain (RBD) which identifies angiotensin-converting enzyme 2 (ACE2) on host cells and mediates the attachment of the virus to ACE2 cellular receptor^16^. Due to the key function of S protein and its RBD in initializing virus entry, extensive studies have been conducted on their mechanisms and structures. For example, Shang et al. provided the crystal structure of the RBD of the S protein in complex with ACE2 and discovered that the binding affinity of RBD to ACE2 was higher in SARS-CoV-2 than that in SARS-CoV due to the unique structural features of SARS-CoV-2^17^. Their team further identified the key mechanism of SARS-CoV-2 cell entry by investigating its receptor binding and protease activation of the spike^18^. A structural analysis of the RBD of the S protein of the SARS-CoV-2 identified the essential residues for its improved binding to ACE2 in comparison with that of SARS-CoV RBD^19^. Further atomic comparison of the conformational variations of the RBD of SARS-CoV-2 was performed to inspect its dynamic features and to guide the intervention strategies for viral entry^20^. The interaction between SARS-CoV-2 Spike protein and ACE2 has been widely investigated through structural analysis and molecular dynamics simulation and several studies showed that the interaction is primarily hydrophobic, as the hydrophobic residues at the ACE2 surfaces contribute significantly to the strong binding between SARS-CoV-2 S protein and ACE2^21–24^.

Bio-layer interferometry (BLI) is a label-free optical analytical technique that analyzes the interference pattern of white light reflected from a biosensor layer with protein immobilized on it and an internal reference layer^25^. When the biosensor is in contact with a solution of the analyte, the analyte binds to the biosensor surface, and the changing thickness of the biosensor layer causes the interference pattern of the reflected light to be shifted. BLI is a useful technique in analyzing protein–protein interactions as it measures binding affinities and rates of associations and dissociation in real time with as little as nanomole of the sample of interest^26^. Over the last decade, BLI has been widely used to investigate antibodies and vaccine development^27–30^. An application of BLI was developed for rapid detection and semi-quantification of SARS-CoV-2 antibodies in plasma samples in less than 20 min using single-use biosensors with automated dip-and-read^31^. As an attempt to develop vaccines during the early stage of COVID-19, a study used BLI to discover a cross-reactive human IgA monoclonal antibody MAb362 which binds to S proteins of SARS-CoV and SARS-CoV-2 and blocks ACE2 receptor binding by overlapping the binding epitope^32^.

A few studies have investigated the stability of SARS-CoV-2 on surfaces in different environmental conditions, and generally the results show that SARS-CoV-2 is more stable at lower temperatures and less persistent on surfaces at higher temperatures^33–37^. However, Kratzel et al. reported no major differences of surface stability over a wide range of temperatures from 4 to 30 °C, challenging the temperature-dependent virus stability suggested otherwise^38^. Currently little is known about the binding behavior and macromolecular interactions of S protein and RBD of SARS-CoV-2 at different conditions—low temperatures, high humidity, and presence of fatty acids—that are typical in critical infrastructures such as meat processing facilities. The objective of this study is to investigate the effect of temperature, fatty acids, ions, and protein concentration on the binding behavior and rates of association and dissociation between the S protein and RBD of SARS-CoV-2 and the hydrophobic aminopropylsilane (APS) biosensors using BLI. The binding to hydrophobic surfaces was investigated due to the hydrophobic nature of ACE2 surface and the ubiquity of hydrophobic surfaces in meat processing plants, as large amounts of fat particles are generated from meat cutting that remain either suspended in the air due to extensive high pressure washing and hosing, or deposited on surfaces. This study provides insight into the binding kinetics rate constants and stability of SARS-CoV-2 S protein and RBD at different environmental conditions and offers explanations why certain combinations of environmental conditions promote the attachment of the virus. Procedures in such environments may be modified based on the outcomes of this study to better protect public health and safety.

## Materials and methods

### Cell growth and purification of RBD and S-2P

Plasmid constructs for transient expression of RBD and S-2P spike proteins in HEK293 cell^39^ were obtained from Dr. Jason McLellan, Dept. of Molecular Biosciences, The University of Texas, Austin. RBD-Fc-8His and S-2P -8His-TwinStrep proteins were produced transiently using Expi293 Expression System following the manufacturer’s (ThermoFisher) recommendations for cultivation and transfection. HEK 293 cells were grown at 37 °C,  ≥ 80% humidity, 8% CO_2_ and shaken at 120 rpm. Four days after transfection, cells were removed by centrifugation at 15,900×*g* for 45 min, and the cell-free culture clarified by a 0.45 µm Polyethersulfone (PES) normal flow filtration.

Clarified cell culture feed was conditioned with 0.2 M NaCl and 10 mM imidazole before the Ni-IMAC metallic immunochromatography step. The adjusted feed material was then filtered through a 0.45 µm PES bottle-top filter membrane prior to loading on a Ni-IMAC FF Sepharose column. The Ni-charged IMAC column was equilibrated with 50 mM sodium phosphate with 300 mM NaCl and 20 mM imidazole, pH 7.4. The clarified feed was loaded onto the column at a linear flow rate of 90 cm/h. The column was washed with 2 column volumes (CVs) of equilibration buffer followed by a wash with 2 CVs of equilibration buffer containing 50 mM imidazole. The bound RBD-8His was eluted with 3 CVs of 50 mM sodium phosphate, 300 mM NaCl buffer containing 250 mM imidazole. All steps except for the sample load were done at linear velocity of 150 cm/h. The imidazole removal from the elution pools was performed using desalting column (HiPrep 26/10 Desalting, Cytiva). Purity assessment of purified proteins was performed by analytical size exclusion chromatography (YMC Pack Diol 200 sizing column) using UV 280 detection.

### Preparation of samples containing SARS-CoV-2 spike protein

The original IMAC (Immobilized Metal Affinity Chromatography) purified, ultrafiltration (UF) concentrated, and diluted by diafiltration (DF) into phosphate-buffered saline (PBS) at a concentration of 0.24 mg/mL SARS-CoV-2 spike (S) Streptavidin and His-tagged recombinant protein, is a highly glycosylated trimer (3 × 158 kDa) with a final weight of about 600 kDa. The S protein was resuspended in PBS to prepare 1:2, 1:5, and 1:10 dilutions at 25 °C. Oleic acid is a common fatty acid naturally occurring in animals and vegetables and is easily accessible. Therefore, oleic acid served as the fatty acid simulant in this study. Oleic acid (Spectrum) was mixed with S protein at 1:1 and 4:1 ratio, respectively, in Eppendorf tubes at 25 °C. The 2 × S protein dilution in PBS was transferred into four separate Eppendorf tubes and (1) placed in ice water to cool to 0 °C, (2) inside a refrigerator to reach 9 °C, (3) at room temperature of 25 °C, and (4) heated up to 37 °C on a heat block. One of the samples at 0 °C was left at the room temperature of 25 °C immediately after it was removed from the ice water.

### Preparation of samples containing SARS-CoV-2 RBD (receptor binding domain)

The purified, original Fc and His-tagged recombinant RBD of SARS-CoV-2 is the binding portion of a single S unit with a size of about 58 kDa. The concentration of the original RBD was 1.822 mg/mL. RBD was diluted in PBS to prepare 1:2, 1:5, and 1:10 dilutions at 25 °C. The four 2 × RBD dilutions were each exposed to different temperatures at 0 °C, 9 °C, 25 °C, and 37 °C similarly to the S protein.

### Determination of the basic kinetics for SARS-CoV-2 spike protein and RBD

Bio-layer interferometry (BLI) was performed to study the basic kinetics of each of the prepared samples of S protein and RBD of SARS-CoV-2 mixed with various substances or exposed to different temperatures. The major instrument used in the analysis was the personal assay BLItz system (ForteBio) with APS biosensors (ForteBio). The APS biosensors were hydrated in PBS in 96 wells plates for at least 10 min before use. Each run started with a baseline step of 30 s with 4 µL PBS as the buffer. After the baseline step, 4 µL S protein or RBD sample at different concentrations or mixed with different substances were added to the drop holder and allowed to associate with the hydrophobic APS biosensor surfaces for 300 s. Following association, APS sensors with attached S protein or RBD were exposed to 4 µL PBS or Milli-Q (MQ) water for 300 s and the rate of dissociation from the APS surfaces was measured. Each sample was analyzed by the BLI at least twice to ensure reproducibility of the results. An APS biosensor and PBS were used in the same manner before each experiment to serve as the reference correction for each assay. The binding curves were generated and fitted to the local model using the BLItz Pro 1.3 Software (ForteBio). The binding affinity, association rate, and dissociation rate were calculated and displayed in the BLItz Pro 1.3 Software (ForteBio). The kinetic constants were shown on the graphs as mean ± standard deviation.

### Statistical analysis

Microsoft Excel 2016 was used to calculate the mean and standard deviation of rates of association and dissociation and to illustrate the data. JMP Pro 16 was used for statistical significance analysis. The one-way Analysis of Variance (ANOVA) was applied to determine the statistical significance among three or more groups. The pooled t-test was used to determine the statistical significance between any two groups. Groups having p-values of less than 0.05 were considered statistically significant differences.

### Protein–ligand interaction verification

The protein crystal structure of the receptor-binding domain complexed with its receptor human ACE2 was obtained from the RCSB protein databank (rcsb.org, PDB: 6VW1)^17,40^. The spike protein was prepared by the Protein Preparation Wizard in Schrödinger suite^41^. The missing side chains were fixed by Prime^42^. The APS sensor molecule was prepared by Chemsketch (Advanced Chemistry Development, Inc.). The interaction information was extracted and refined by the Enhanced Ligand Exploration and Interaction Recognition Algorithm (ELIXIR-A) platform^43^. Molecular docking was performed using the software Autodock vina with the default protocol^44^. The protein–ligand interactions were analyzed in Maestro^45^.

### Molecular dynamics (MD) simulation

The protein structure was obtained from RCSB protein databank (PDB: 6VW1) and prepared by the Protein Preparation Wizard in Schrödinger suite. The structure of oleic acid was obtained from ZINC15 database (ZINC6845860)^46^ and neutralized using LigPrep^47^. The MD simulation was performed using Desmond^48^. Desmond’s System Builder was used to generate a solvated system in an orthorhombic simulation box with absolute distance of 80 Å × 80 Å × 80 Å. The solvent model was transferable intermolecular potential with 3 points (TIP3P). The system was neutralized with sodium or chloride ions and 0.1 M sodium chloride was added. The first step was Desmond's standard relaxation protocol under isothermal–isobaric ensemble. And the force field was OPLS3e^49^. The full simulation was maintained at temperature of 300 K and pressure of 1 atm for 5 ns period. The recording frequency was 20 ps, 250 frames in total, excluding the initial reference frame. The hydrophobicity analysis was performed with Hydrophobic/philic Surfaces Panel. The cut-off particular potential value (isovalue) for the hydrophilic and hydrophobic regions are − 6 and − 0.5 kcal/mol, respectively.

## Results and discussion

### S protein diluted in PBS dissociating in PBS and in water at 25 °C; 1.569 µM S protein dissociating in water at five temperatures

**Figure 1:**
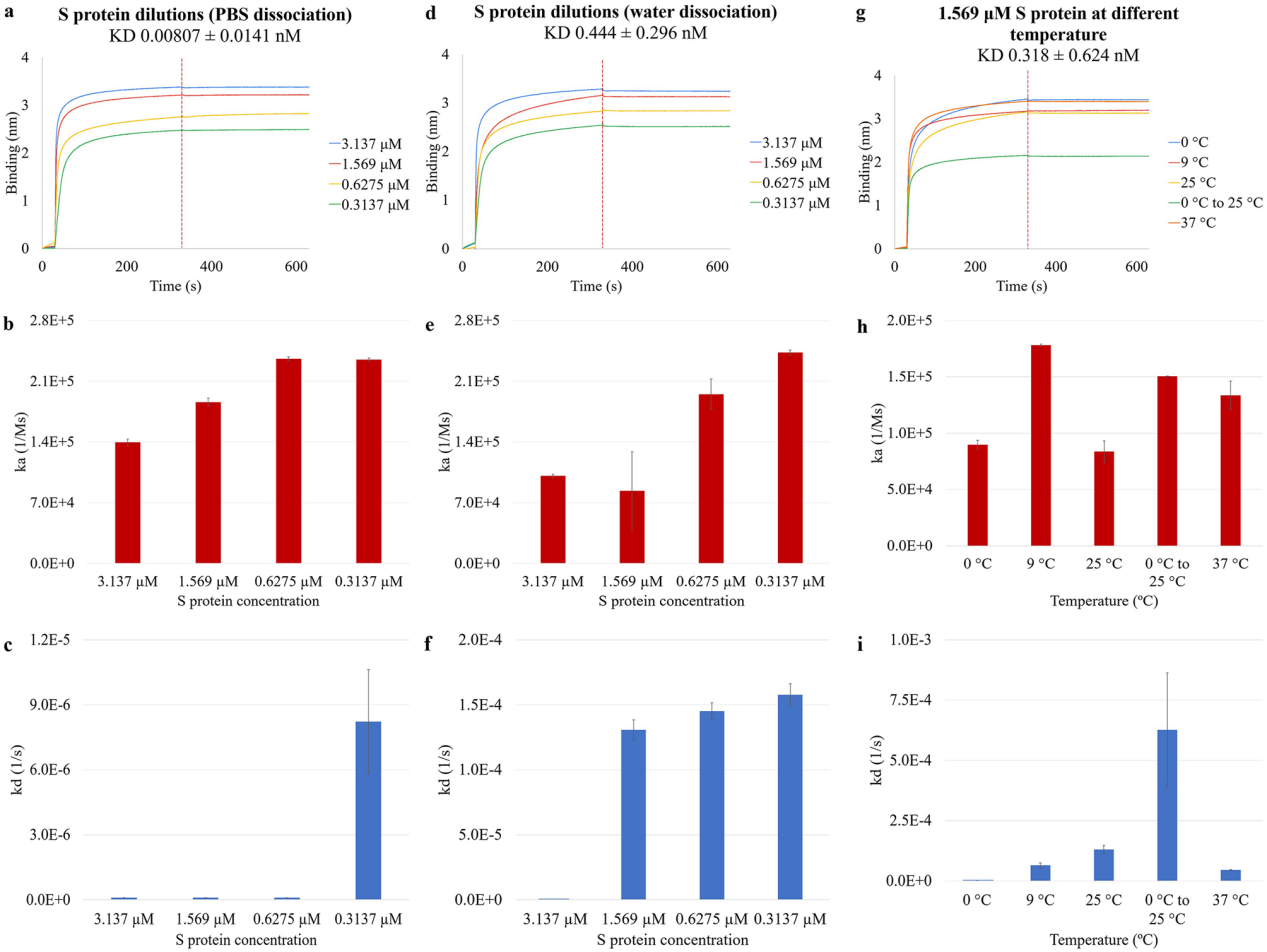
The effects of protein concentrations, ionic PBS dissociation, water dissociation, and temperature on the attachment and detachment of S protein to and from the hydrophobic APS sensors. The binding curves were generated from the basic kinetics analysis of 3.137 µM, 1.569 µM, 0.6275 µM, and 0.3137 µM S protein dissociating (**a**) in PBS and (**d**) in purified water, and (**g**) 1.569 µM S protein at 0 °C, 9 °C, 25 °C, 0 °C to 25 °C, and 37 °C dissociating in water. The vertical dotted line on the figures indicates the end of the association or the start of the dissociation step. The association constant ka was shown for the four S protein concentrations dissociating (**b**) in PBS and (**e**) in water, and (**h**) for 1.569 µM S protein at 0 °C, 9 °C, 25 °C, 0 °C to 25 °C, and 37 °C dissociating in water. The dissociation constant kd was shown in the same order for the three groups of conditions in (**c**), (**f**), and (**i**).

Based on the concentration and the molecular weight of the original S protein, the molar concentrations for the 1:1 (1x), 1:2 (2x), 1:5 (5x), and 1:10 (10x) PBS diluted S protein were computed in the BLI system to be 3.137 µM, 1.569 µM, 0.6275 µM, and 0.3137 µM. The first set of basic kinetics analysis was performed with the APS sensors dissociating in PBS at room temperature of 25 °C. The strongest binding was observed with 3.137 µM S protein forming a binding layer of 3.39 nm, and the most diluted S protein showed the thinnest binding layer on the sensor of 2.50 nm out of the four dilutions (Fig. 1a). The analysis indicates that the binding activities were stronger with a higher concentration of S protein present in the sample. At the end of the association step, the thickness of the binding layer for 3.137 µM, 1.569 µM, 0.6275 µM, and 0.3137 µM S protein were 3.39 nm, 3.21 nm, 2.76 nm, and 2.48 nm, respectively. For all the four binding curves, the values increased greatly at the beginning of the association step, then became unchanged for the rest of the analysis period. The association constants (ka) of 3.137 µM, 1.569 µM, 0.6275 µM, and 0.3137 µM S protein were 1.40E5 Ms^−1^, 1.86E5 Ms^−1^, 2.36E5 Ms^−1^, and 2.35E5 Ms^−1^, respectively. These results in Fig. 1b show that decreasing S protein concentration from 3.137 µM to 0.6275 µM led to increasing association constants, as the less concentrated S protein bound to the hydrophobic surface at a higher rate; the difference between the ka at the four concentrations was statistically significant (p = 0.0001). Further diluting of S protein from 0.6275 µM to 0.3137 µM had minimal effect on the ka. Dissociation constant kd measures how fast proteins detach from the biosensor. The dissociation constants (kd) of 3.137 µM, 1.569 µM, 0.6275 µM, and 0.3137 µM S protein were 1.00E−7 s^−1^, 1.00E−7 s^−1^, 1.00E−7 s^−1^, and 8.22E−6 s^−1^, respectively. Figure 1c shows some but still low dissociation in the presence of ions in PBS from the hydrophobic surface only at the lower protein concentration of 0.3137 µM. All the other three S protein concentrations had minimal to no detachment, meaning that the S protein was not able to resuspend into the ionic environment once it bound to the hydrophobic surface. However, the kd for the four S protein concentrations was not statistically different (p = 0.1843).

The basic kinetics analysis was repeated on 3.137 µM, 1.569 µM, 0.6275 µM, and 0.3137 µM S protein with performing the dissociation step with purified water instead of PBS to investigate the role of ions in S protein detachment. The binding curves shown in Fig. 1d shared the similar features as Fig. 1a, with the most concentrated S protein generating the thickest binding layer throughout the analysis. At the end of association, the binding layers for 3.137 µM, 1.569 µM, 0.6275 µM, and 0.3137 µM S protein were 3.29 nm, 3.16 nm, 2.84 nm, and 2.54 nm, respectively. The steep increase in the binding layer indicates that the majority of the binding occurred at the beginning of the association step (Fig. 1d). The ka of 3.137 µM, 1.569 µM, 0.6275 µM, and 0.3137 µM S protein dissociating in water were 1.01E5 Ms^−1^, 8.36E4 Ms^−1^, 1.95E5 Ms^−1^, and 2.43E5 Ms^−1^, respectively. From Fig. 1e, the association pattern is similar to the previous behavior in the ionic environment as the least concentrated S protein shows the highest association rate, and the difference between the ka of four concentrations was also statistically significant (p = 0.0011). Although the ka of the 1.569 µM S protein was lower than the 3.137 µM S protein, the difference was small (within one magnitude) and was not statistically significant (p = 0.3089). Therefore, the same observation as in Fig. 1b that in general decreasing S protein concentrations resulted in increasing ka is still relevant. The kd of 3.137 µM, 1.569 µM, 0.6275 µM, and 0.3137 µM S protein concentrations were 1.00E−7 s^−1^, 1.31E−4 s^−1^, 1.45E−4 s^−1^, and 1.58E−4 s^−1^, respectively. Figure 1f shows that dissociation from the hydrophobic surface occurred at higher protein concentrations of 1.569 µM when ions were not present in the environment, with decreasing S protein concentrations showing increasing dissociation within a narrow kd range; the difference between the four ka values was statistically significant (p = 0.0029). The results from Fig. 1c (dissociation in PBS) and Fig. 1f (dissociation in water) show that the ionic environment enhanced the attachment of S protein to the hydrophobic surface, making it more difficult for the already attached S protein to resuspend into the solution.

To investigate the effect of temperature on the binding of S protein to the hydrophobic APS surface, 1.569 µM S protein at four temperatures—0 °C, 9 °C, 25 °C, and 37 °C—was studied. In addition, 1.569 µM S protein that was previously kept at 0 °C was transferred to room temperature of 25 °C and was compared with the S protein at the same concentration without freezing. This set of experiments was designed to answer the question whether freezing can induce change on the binding of S protein. From Fig. 1g, the binding curves of 1.569 µM S protein at 37 °C and 9 °C showed similar behaviors, as they both bound quickly at the beginning of the association step and the binding became much slower during the rest of the period. On the other hand, the binding of 1.569 µM S protein at 0 °C and 25 °C was slower at the beginning and faster for the rest of the association step, resulting in a continuously increasing curve. The binding curve of the 0 °C to 25 °C 1.569 µM S protein had the similar shape as 37 °C and 9 °C, but with weaker binding activities overall. Although behaviors during association were not all the same for the five temperatures, all the binding distance remained relatively constant during the dissociation step, indicating minimal resuspension of S protein into water from the APS sensor surface. At the end of the association, the binding layers for 1.569 µM S protein at 0 °C, 9 °C, 25 °C, 0 °C to 25 °C, and 37 °C were 3.46 nm, 3.17 nm, 3.16 nm, 2.16 nm, and 3.41 nm, respectively. The ka of 1.569 µM S protein at 0 °C, 9 °C, 25 °C, 0 °C to 25 °C, and 37 °C were 8.96E4 Ms^−1^, 1.78E5 Ms^−1^, 8.36E4 Ms^−1^, 1.50E5 Ms^−1^, and 1.34E5 Ms^−1^, respectively. From Fig. 1h, 1.569 µM S protein at 0 °C and 25 °C shows a lower rate of association while the 9 °C test shows the highest association. The ka among the S protein at four temperatures—0 °C, 9 °C, 25 °C, and 37 °C—was statistically significantly different (p = 0.006), indicating that temperature impacts the rate of association of 1.569 µM S protein. The ka at 9 °C was significantly higher than that at 0 °C (p = 0.004) and 25 °C (p = 0.007). The highest ka at 9 °C in our study is consistent with the results of Shi et al. stating that the number of COVID-19 cases was the highest when the temperature was at around 10 °C and can be correlated to the higher numbers of infectious cases found in their study^50^. The kd of 1.569 µM S protein at 0 °C, 9 °C, 25 °C, 0 °C to 25 °C, and 37 °C were 2.26E−7 s^−1^, 6.44E−5 s^−1^, 1.31E−4 s^−1^, 6.27E−4 s^−1^, and 4.52E−5 s^−1^, respectively. 1.569 µM S protein at 0 °C, 9 °C, 25 °C, and 37 °C shows significantly different (p = 0.0066) but low rate of dissociation, indicating strong attachment of the S protein to the hydrophobic surface, especially at 0 °C (Fig. 1i). The kd at 0 °C was significantly lower than those at the higher temperatures of 9 °C (p = 0.0433), 25 °C (p = 0.0126), and 37 °C (p = 0.0043). The low kd value at 0 °C shows strong attachment between the S protein and the hydrophobic surface, indicating high stability. This finding is in agreement with the conclusion of Chin et al. that the virus was highly stable at low temperature at around 4 °C^51^. These results suggest that the S protein is less likely to resuspend from the hydrophobic surface, such as the high amount of fat generated during meat processing, at 0 °C due to the strong attachment. At temperature as low as 0 °C which is commonly found in the chiller rooms of meat processing plants, S protein is more likely to stay entrained in the airflow for longer periods of time once they form the strong attachment to the aerosolized fats. Figure 1i also shows that exposing the 1.569 µM S protein to a wide temperature range from 0 to 25 °C resulted in strong protein detachment. Comparing the two 1.569 µM S protein at 25 °C, the one with previous freezing at 0 °C had higher rate of dissociation than the one without freezing, yet the difference was not significant (p = 0.1407). The previously frozen 25 °C S protein also had significantly (p = 0.0183) higher rate of association as shown in Fig. 1h. This indicates that freezing can cause change in S protein that affects its binding kinetics at higher temperatures.

### S protein diluted in oleic acid, dissociating in water at 25 °C

**Figure 2:**
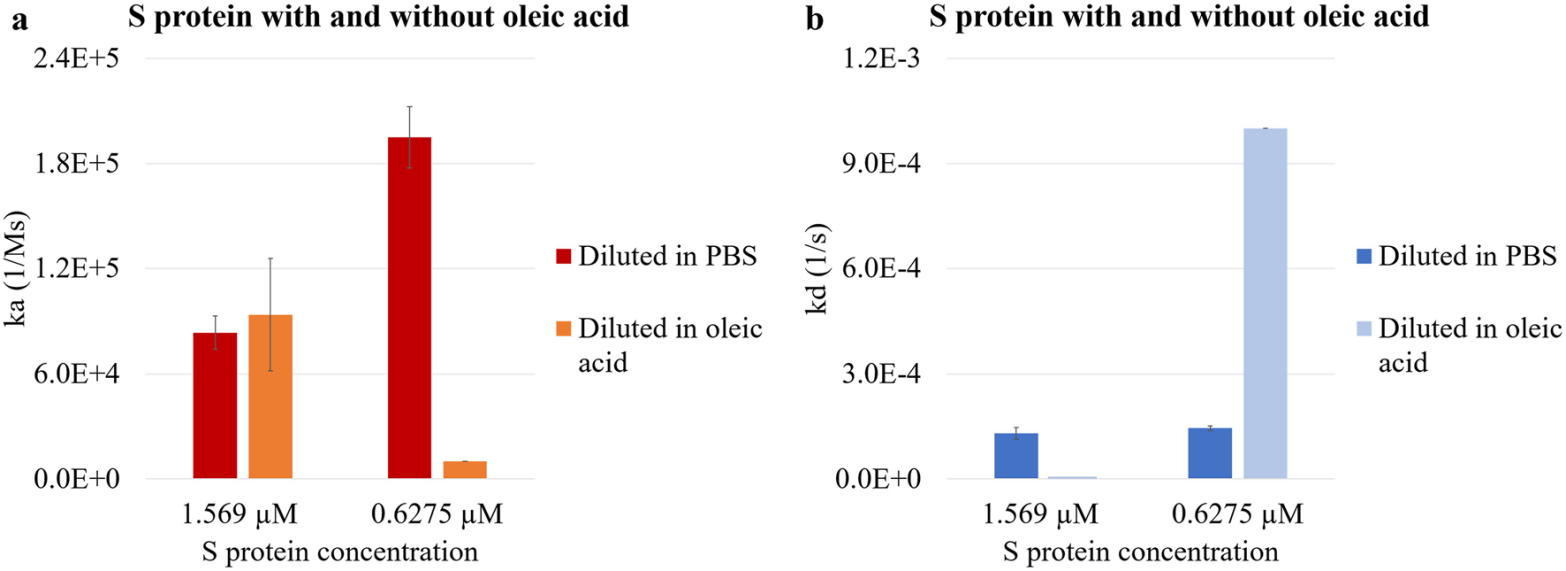
The effects of oleic acid on the attachment and detachment of S protein to and from the hydrophobic APS sensors. (**a**) The association constant ka and (**b**) dissociation constant kd of PBS diluted and oleic acid diluted S protein at 1.569 µM and 0.6275 µM.

Oleic acid was used to dilute S protein to 1.569 µM and 0.6275 µM. The oleic acid and S protein mixtures underwent the basic kinetic analysis to obtain their kinetic profiles. The ka values of 1.569 µM and 0.6275 µM S protein in oleic acid were 9.37E4 Ms^−1^ and 1.00E4 Ms^−1^. Figure 2a shows that decreasing S protein concentration (1.569 µM and 0.6275 µM) in oleic acid results in decreasing rate of association, yet the difference was not statistically significant (p = 0.0652). The difference between the ka of the PBS diluted and oleic acid diluted S protein at 0.6275 µM was statistically significant (p = 0.0178). The kd values of 1.569 µM and 0.6275 µM S protein in oleic acid were 1.00E−7 s^−1^ and 1.00E−3 s^−1^. From Fig. 2b, only minimal dissociation was recorded at higher S protein concentration of 1.569 µM in oleic acid, while the kd was significantly higher with the 0.6275 µM S protein (p = 0.0099). When comparing the kd of PBS diluted S protein and the oleic acid diluted S protein at the same protein concentrations, the differences were statistically significant at both the 1.569 µM S protein (p = 0.0125) and the 0.6275 µM S protein (p = 0.0001). During meat processing high amounts of fats and lipids are generated and become aerosolized, allowing it to remain in the air. Our results suggest that the presence of oleic acid in the environment changes the attachment and detachment between S protein and the hydrophobic surface. Lipids play an important role in virus binding. Shoemark et al. showed that linoleate—an ester of the fatty acid linoleic acid—stabilized the locked conformation of the S protein, and, on the other hand, cholesterol—a lipid—destabilized the closed structure, indicating that both substances can affect the structure and binding capabilities of the S protein^52^. The same stabilizing effect of binding linoleic acids was proven by Toelzer et al. using cryo-electron microscopy, and the linoleic acid binding was determined to be irreversible^53,54^. Furthermore, dynamical-nonequilibrium simulation results showed that the fatty acid binding site of the S protein can affect functionally important sites that are distant^55^. Another study stated that loss of lipids can destabilize the trimeric S protein and prevent receptor binding, however, the infectivity of released virus can be impacted by the nature of lipids that are present at the site of infection^56^. Our results show that the 1.569 µM S protein in oleic acid had smaller kd due to stronger attachment to the hydrophobic APS surface compared to the 0.6275 µM S protein in oleic acid, indicating that the S protein at higher concentrations may undergo conformational changes in fatty acids. Our results suggest that, in addition to the ligands already studied by Shoemark et al. and Carrique et al., oleic acid can also affect the attachment and detachment of S protein from the hydrophobic surface, which is in agreement with their findings, and this impact is concentration dependent. Future studies need to be conducted to investigate the scope of this impact.

### RBD diluted in PBS dissociating in PBS and in water at 25 °C; 13.4 µM RBD dissociating in water at five temperatures

**Figure 3:**
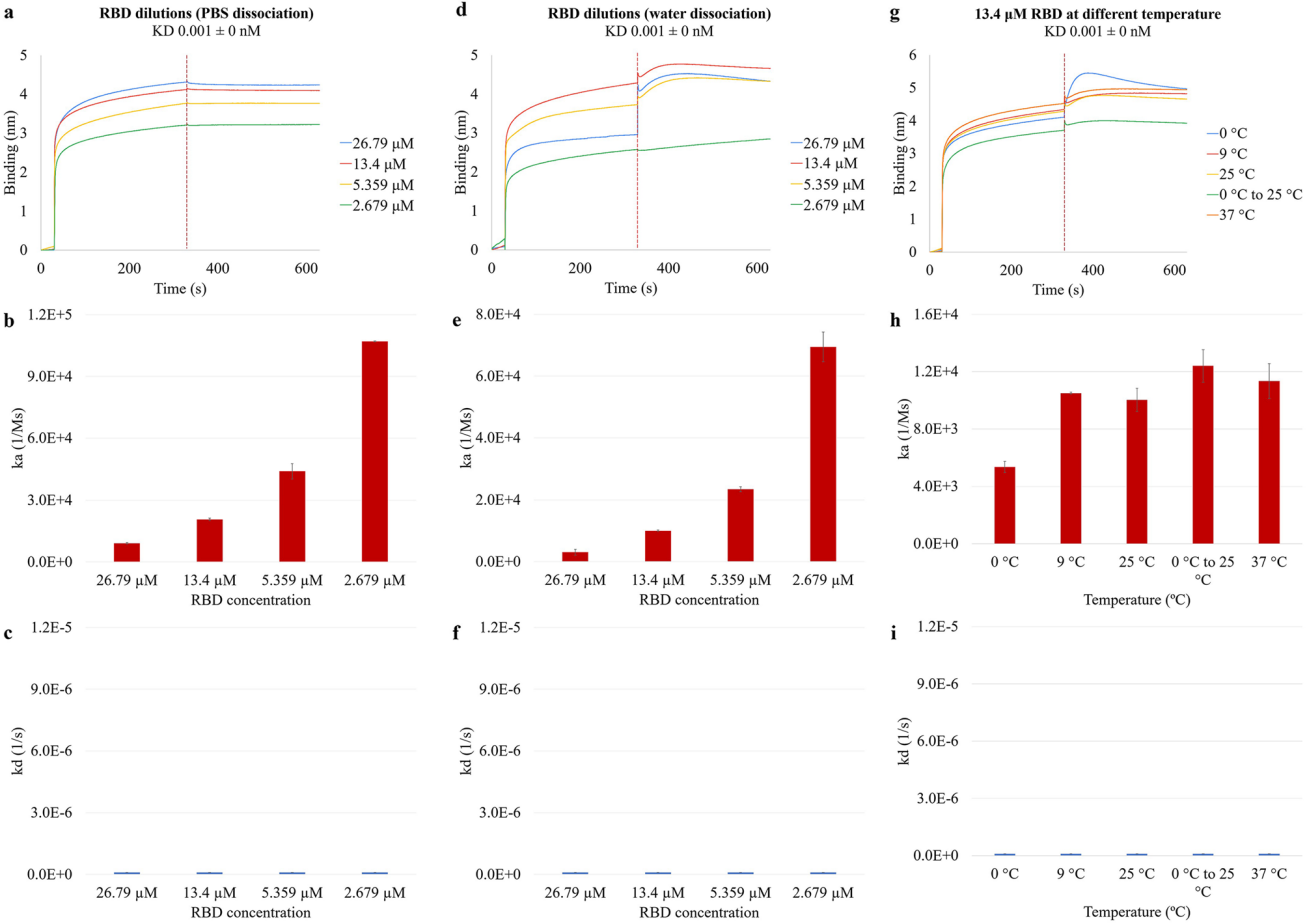
The effects of protein concentrations, ionic PBS dissociation, water dissociation, and temperature on the attachment and detachment of RBD to and from the hydrophobic APS sensors. The binding curves were generated from the basic kinetics analysis of 26.79 µM, 13.4 µM, 5.359 µM, and 2.679 µM RBD dissociating (**a**) in PBS and (**d**) in purified water, and (**g**) 13.4 µM RBD at 0 °C, 9 °C, 25 °C, 0 °C to 25 °C, and 37 °C dissociating in water. The vertical dotted line on the figures indicates the end of the association or the start of the dissociation step. The association constant ka was shown for the four S protein concentrations dissociating (**b**) in PBS and (**e**) in water, and (**h**) for 13.4 µM RBD at 0 °C, 9 °C, 25 °C, 0 °C to 25 °C, and 37 °C dissociating in water. The dissociation constant kd was shown in the same order for the three groups of conditions in (**c**), (**f**), and (**i**).

The binding kinetics of purified RBD was studied and compared to that of the S protein. Both the S protein and RBD are important proteins of the SARS-CoV-2 and were the major focus of related studies. The S protein contains not only the RBD, but other domains and structural units including an N-terminal domain (NTD), C-terminus, fusion peptide (FP), heptad repeat 1 and 2 (HR1 and HR2). The RBD can only bind to the ACE2 receptor when it is in the up state, in contrast to when it is partially buried in the down state^18,57^. RBD of SARS-CoV-2 was diluted in PBS to reach molar concentration of 26.79 µM, 13.4 µM, 5.359 µM, and 2.679 µM. The basic kinetics analysis was performed with APS sensors at 25 °C with water dissociation. The binding curve was shown in Fig. 3a. At the end of the association, the thickness of the binding layer for 26.79 µM, 13.4 µM, 5.359 µM, and 2.679 µM were 4.31 nm, 4.12 nm, 3.77 nm, and 3.21 nm, respectively. Similar to S protein dilutions in Fig. 1a, the strongest binding activities were observed with the most concentrated 26.79 µM RBD dilution. Weaker binding curves were obtained with the less concentrated RBD. All the RBD dilutions showed similar behavior throughout the analysis. The RBD bound to the hydrophobic surface rapidly at the beginning of the association, and the binding rate slowed down as the analysis proceeded. Unlike the kinetics profile of S protein dilutions in Fig. 1a, the binding layer of RBD dilutions continuously grew during the entire association step, while S protein dilutions reached the maximum binding capacity in a short time period. For all the four RBD dilutions, the binding curve stayed plateau during the dissociation step. The ka values of 26.79 µM, 13.4 µM, 5.359 µM, and 2.679 µM were 9.09E3 Ms^−1^, 2.07E4 Ms^−1^, 4.40E4 Ms^−1^, and 1.07E5 Ms^−1^, respectively. Similar to the S protein, decreasing RBD concentrations show increasing rate of association (Fig. 3b), and the difference of the ka among the four concentrations was statistically significant (p = 0.0001). The kd values of 26.79 µM, 13.4 µM, 5.359 µM, and 2.679 µM were 1.00E−7 s^−1^, 1.00E−7 s^−1^, 1.00E−7 s^−1^, and 1.00E−7 s^−1^, respectively. The results from Fig. 3c showed that uniformly minimal or no dissociation from the hydrophobic surface was recorded for all four concentrations.

The basic kinetics analysis was performed with 26.79 µM, 13.4 µM, 5.359 µM, and 2.679 µM RBD dilutions at 25 °C with water dissociation. Figure 3d shows that all the four RBD dilutions bound rapidly to the APS sensor at the beginning of the association step, and the binding rate became slower for the rest of the step. The binding curve for 26.79 µM RBD barely increased after the rapid binding period, while the other three RBD dilutions continuously bound at a slow rate. At the end of the association, the thickness of the binding layers for 26.79 µM, 13.4 µM, 5.359 µM, and 2.679 µM RBD dilutions were 2.96 nm, 4.29 nm, 3.73 nm, and 2.58 nm, respectively. The rapid increase at the start of the dissociation step was due to random errors in the BLItz system and should not be counted as part of the binding layer. During the dissociation step, 26.79 µM, 13.4 µM, and 5.359 µM RBD dilutions continuously bound to the APS sensor, the binding layers then decreased during the rest of the dissociation step as part of it re-suspended into water. The binding for 2.679 µM RBD dilution increased steadily during the entire dissociation step. The ka of 26.79 µM, 13.4 µM, 5.359 µM, and 2.679 µM were 3.05E3 Ms^−1^, 1.00E4 Ms^−1^, 2.35E4 Ms^−1^, and 6.95E4 Ms^−1^, respectively. The kd of 26.79 µM, 13.4 µM, 5.359 µM, and 2.679 µM RBD dilutions were 1.00E−7 s^−1^, 1.00E−7 s^−1^, 1.00E−7 s^−1^, and 1.00E−7 s^−1^, respectively. Figure 3e demonstrates a similar trend as in Fig. 3b that decreasing RBD concentrations corresponded to increasing association, and the difference was statistically significant (p = 0.0002). The same uniformly minimal or no dissociation from the hydrophobic surface was shown in Fig. 3f. Compared to the S protein, the ionic environment provided with PBS had less impact on both the ka and kd of the RBD at all levels of protein concentrations.

The 13.4 µM RBD was studied at five temperature conditions—0 °C, 9 °C, 25 °C, 0 °C to 25 °C, and 37 °C. The 13.4 µM RBD at 37 °C showed stronger binding activities compared to the other four temperatures (Fig. 3g). All the five binding curves increased rapidly at the beginning of the association and slower for the rest of the step. At the end of association, the thickness of the binding layers for 13.4 µM RBD at 0 °C, 9 °C, 25 °C, 0 °C to 25 °C, and 37 °C were 4.11 nm, 4.34 nm, 4.29 nm, 3.71 nm, and 4.53 nm, respectively. At all the five temperatures the binding layer continuously grew for a short period during the dissociation step, although 13.4 µM RBD at 0 °C to 25 °C showed less binding compared to the other concentrations. After reaching the maximum binding layers, all the binding curves decreased gradually as the attached RBD resuspended into water. The ka of 13.4 µM RBD at 0 °C, 9 °C, 25 °C, 0 °C to 25 °C, and 37 °C were 5.33E3 Ms^−1^, 1.05E4 Ms^−1^, 1.00E4 Ms^−1^, 1.24E4 Ms^−1^, and 1.13E4 Ms^−1^, respectively. From Fig. 3h, out of the four temperatures—0 °C, 9 °C, 25 °C, and 37 °C—the 13.4 µM RBD at 0 °C shows the lowest rate of association and the 37 °C test shows the highest ka. The difference of ka among 0 °C, 9 °C, 25 °C, and 37 °C was statistically significant (p = 0.0383), although no general trend was observed over the tested temperature range. The 25 °C 13.4 µM RBD with previous freezing had a higher ka value than the one without freezing, yet the difference is not statistically significant (p = 0.1092). The kd of 13.4 µM RBD at 0 °C, 9 °C, 25 °C, 0 °C to 25 °C, and 37 °C were 1.00E−7 s^−1^, 1.00E−7 s^−1^, 1.00E−7 s^−1^, 1.00E−7 s^−1^, and 1.00E−7 s^−1^, respectively. Figure 3i shows uniformly minimal or no dissociation from the hydrophobic surface at all the tested temperatures. Compared to S protein where temperature impacted the kd significantly and a wide temperature change from 0 to 25 °C induced strong protein detachment, changing the temperature of the environments had less effects on the resuspension of RBD.

### S protein without tags diluted in PBS, dissociating in PBS at 25 °C

The recombinant S protein was further purified to remove Streptavidin and His tags and underwent BLI to confirm that the interferometry results in the previous sections were not significantly affected by the tags and can be applied to S protein without tags. The truncated S protein was diluted in PBS to obtain the same molar concentrations used for the recombinant S protein of 3.137 µM, 1.569 µM, 0.6275 µM, and 0.3137 µM. The baseline and dissociation steps were established with PBS. The BLI analysis of the four dilutions was performed at a constant room temperature of 25 °C.

**Figure 4:**
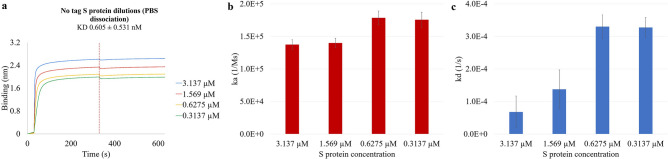
The effects of protein concentrations on the attachment and detachment of the no-tagged S protein to and from the hydrophobic APS sensors. The Streptavidin and His tags were removed from the recombinant S protein to confirm that the interferometry results in the previous sections were not significantly affected by the tags (p = 0.8304 for ka and p = 0.2947 for kd) and the findings can be applied to S protein without tags. (**a**) The binding curves were generated from the basic kinetics analysis of 3.137 µM, 1.569 µM, 0.6275 µM, and 0.3137 µM no-tagged S protein dissociating in PBS. The vertical dotted line on the figures indicates the end of the association or the start of the dissociation step. The association constant ka and the dissociation constant kd were shown for the four no-tagged S protein concentrations dissociating in PBS in (**b**) and (**c**), respectively.

The thickest binding layer was formed with the most concentrated no-tagged S protein at 3.137 µM, and the thinnest binding layer was found with the least concentrated no-tagged S protein of 0.3137 µM (Fig. 4a). These results are in agreement with the S protein binding curves in Fig. 1a. The interferometry results suggest the similar positive correlation between the concentrations of S protein and the amounts of ligands binding to the APS sensors, meaning that increasing S protein concentration in the solution leads to stronger binding. At the end of the association, the thickness of the binding layers for 3.137 µM, 1.569 µM, 0.6275 µM, and 0.3137 µM no-tagged S protein were 2.61 nm, 2.34 nm, 2.09 nm, and 1.99 nm, respectively. The shape of the binding curves in Fig. 4a was similar to Fig. 1a, with rapid ligand binding to the sensor at the beginning of the association and minimum to none change for the rest of the analysis. The ka of 3.137 µM, 1.569 µM, 0.6275 µM, and 0.3137 µM no-tagged S protein were 1.37E5 Ms^−1^, 1.40E5 Ms^−1^, 1.79E5 Ms^−1^, and 1.76E5 Ms^−1^, respectively. Comparing with Fig. 1b, the no-tagged and tagged S protein had ka values at the same magnitude of 1E5 Ms^−1^. The ka values for all four concentrations varied within a small range, with the more concentrated S protein having higher ka values; the difference of ka among the four concentrations was statistically significant (p = 0.032). The kd of 3.137 µM, 1.569 µM, 0.6275 µM, and 0.3137 µM S protein were 6.82E−5 s^−1^, 1.38E−4 s^−1^, 3.30E−4 s^−1^, and 3.28E−4 s^−1^, respectively. Compared to the recombinant S protein, the kd for the no-tagged S protein was in general two magnitudes higher, with the smallest kd recorded with the highest concentration of 3.137 µM (Fig. 4c). The difference of kd among the four tested concentrations was statistically significant (p = 0.014). Although higher kd was obtained with the no-tagged S protein, the kd was still low, indicating minimum detachment from the hydrophobic APS surface at all tested protein concentrations. When comparing the ka and kd of the no-tagged and recombinant S protein at the same concentration of 3.137 µM, the difference was neither statistically significant for the ka (p = 0.8304) nor for the kd (p = 0.2947). Since the no-tagged S protein shared similar ka and higher but still small kd as the recombinant S protein, it is proved that the tags on the recombinant S protein did not significantly affect the binding affinities to the APS sensors, and the interferometry results of recombinant S protein can apply to no-tagged S protein.

### Protein–ligand interaction verification

**Figure 5:**
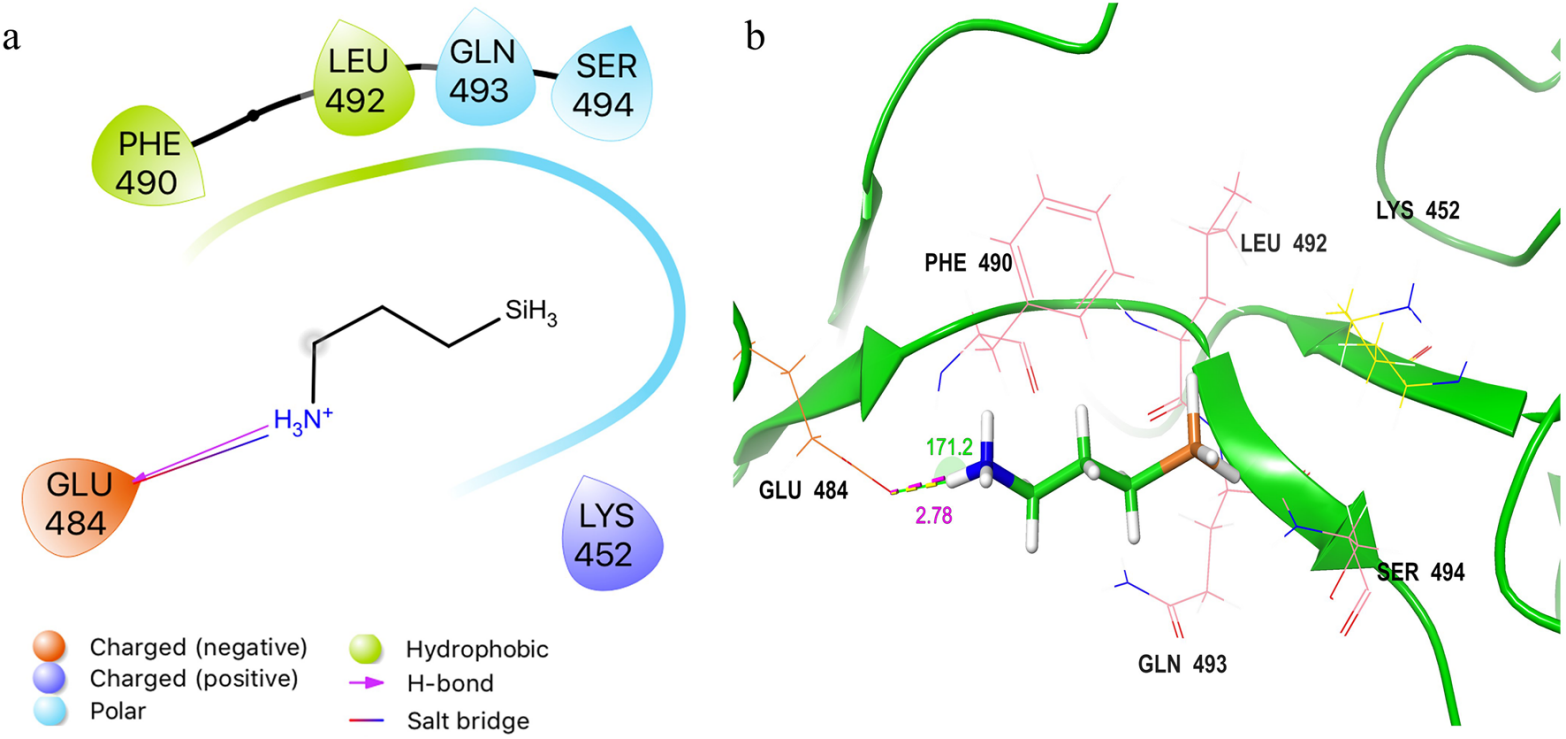
The 2D (**a**) and 3D (**b**) interaction diagrams of the docked APS ligand against the RBD domain of the spike protein. Interacted residues were labeled. The hydrogen bond is shown in purple.

The docking pose of the APS ligand to the RBD shows the formation of multiple non-covalent bonds (Fig. 5a). One hydrogen bond and one salt bridge were found between APS and GLU484. The distance of the hydrogen bond was 2.78 Å and the donor-hydrogen-acceptor angle was 171.2° (Fig. 5b). Two hydrophobic bonds were formed with residues PHE490 and LEU492. Two polar interactions were formed with residues GLN493 and SER494. An ionic bond was formed at residue LYS452. This single APS gave a binding affinity of − 1.834 kcal/mol due to the limited binding capacity of the tiny ligands. However, the complex of ligands and proteins showed good close contacts, suggesting that immobilized APS arrays can produce strong binding interactions with RBD. The presence of hydrophobic and ionic residues in the interaction was also in agreement with the results of interferometry of BLI.

### Molecular dynamics (MD) simulation of RBD with fatty acids

**Figure 6:**
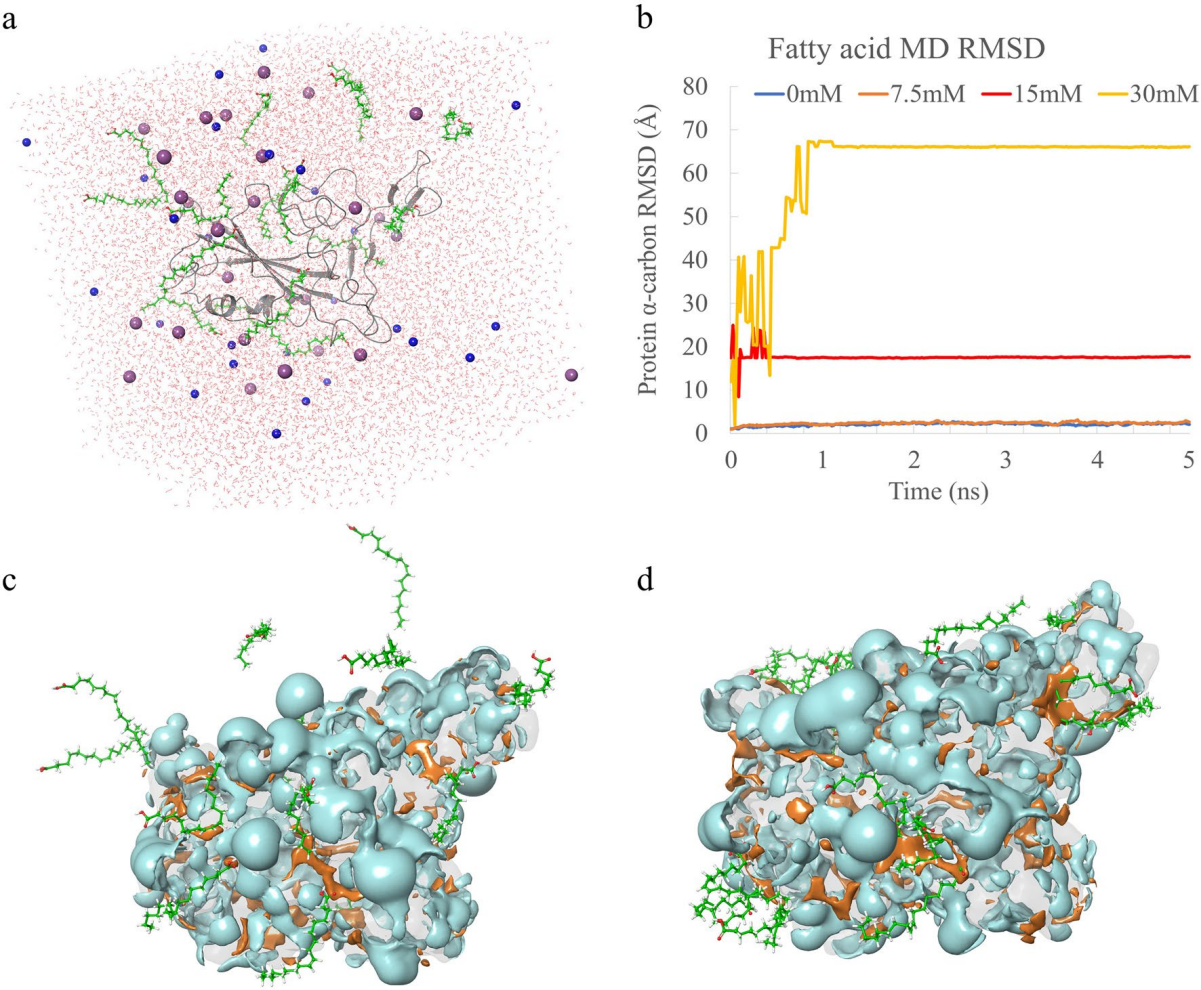
(**a**) Snapshot of initial frame of RBD (gray) with 30 mM fatty acid (green) in solvent model. TIP3P water models were red, sodium ions were blue, and chloride ions were purple. (**b**) RMSD plots of RBD with different concentrations of fatty acids. Initial snapshot (**c**) and final snapshot (**d**) of RBD hydrophobic (orange) and hydrophilic (cyan) surfaces with 30 mM fatty acid (green).

To understand the effect of fatty acids on RBD at certain temperatures and ionic concentrations in the aqueous solvents, all-atom MD simulations in explicit solvent models were performed (Fig. 6a). In the solvent model, 0.1 M Na^+^ and Cl^−^ ions were added. Four levels of fatty acid concentrations were tested (0 mM, 7.5 mM, 15 mM and 30 mM) under temperature of 300 K and pressure of 1 atm. In Fig. 6b, RBD protein α-carbon root-mean-square deviation (RMSD) values were monitored throughout the simulation. RMSD is related to the stability of protein structure, and high RMSD values (> 3 Å) indicate that the structure of the protein may have changed during the simulation. In Table 1, the RMSD was increased with the increase of fatty acid concentration. In the control group, the RMSD was 2.08 ± 0.31 Å, suggesting a stable simulation. While, in the group of 30 mM fatty acid, the RMSD increased to 61.40 ± 12.33 Å. In high concentrations of fatty acids, the RBD structure was changed. The structural change occurred in the first 1 ns, and then the fluctuations decreased to a low level (Fig. 6b). The RMSD value of 66.07 ± 0.07 Å for the second half of the 30 mM simulation also confirmed this observation. The standard deviation decreased from 12.33 to 0.07 Å. The same results were found in simulations of the other concentrations. These results confirmed that all four groups of MD simulations ended up at equilibrium.

**Table 1:** RMSD and surface area of the MD simulation.

Fatty acid (mM)	0	7.5	15	30
**RMSD (Å)**				
Full simulation	2.08 ± 0.31	2.35 ± 0.30	17.63 ± 1.10	61.40 ± 12.33
Second half simulation	2.17 ± 0.18	2.48 ± 0.19	17.57 ± 0.07	66.07 ± 0.07
**Surface area (Å**^**2**^**)**				
Hydrophilic	10,534.922	10,198.55	10,500.254	10,123.745
Hydrophobic	1812.358	1844.913	1972.944	2053.426

In Fig. 6c,d, the fatty acid molecules were aggregated after MD simulation. Most of the fatty acid molecules were attached to the hydrophobic surface. The hydrophobic surface area of the RBD increased with the increase of fatty acids. In the control group, hydrophobic surface area was 1812.358 Å^2^, while in the group of 30 mM fatty acid, the hydrophobic surface area was increased to 2053.426 Å^2^. Hydrophobic residues inside the protein came into contact with fatty acids, thereby increasing the hydrophobic surface of the RBD. Meanwhile, the hydrophobic region was occupied by fatty acids, which may reduce the binding ability of RBD proteins.

## Conclusion

In this study, three conditions are identified to enhance the attachment of the purified S protein and its RBD to hydrophobic surfaces: high ionic concentration, presence of hydrophobic fatty acids, and low temperature. The S protein exposed to a wide temperature change from 0 °C to 25 °C within one hour results in S protein detachment, suggesting that freezing can cause structural changes in the S protein that affect its binding kinetics after it is recovered at higher temperature. This rapid change of temperature within an hour was applied to simulate the sudden temperature drop which is common in meat processing plants when the workers move between warmer and colder locations in the facility, for example, from the 25 °C breakroom to the chiller or fabrication rooms where temperature is kept low, usually < 12 °C, and even lower due to the presence of dry ice containers to keep the products safe during processing. As virus aerosols can also be transported with the airflow through the openings between these locations, they become exposed to the different temperatures. At all the conditions, RBD exhibits lower dissociation capabilities than the full-length S trimer protein, indicating that the separated RBD formed stronger attachment to hydrophobic surfaces compared to when it was included in the S protein. The interaction between RBD of S protein and APS ligand was verified via molecular docking. MD simulation further revealed that the presence of fatty acid molecules has the potential to increase the hydrophobic surface area of RBD, changing its binding ability. The findings of this study implied that certain environmental conditions—low temperature, high humidity, and presence of fatty acids—that are typical in critical infrastructures such as meat processing plants enhance the binding by the S protein and RBD of the SARS-CoV-2 to hydrophobic surfaces. Under such conditions, SARS-CoV-2 is harder to be removed through typical sanitation procedures such as ventilation and hosing due to the enhanced attachment. The findings also suggested that the environmental conditions affect the transmission of SARS-CoV-2. With the presence of fat particles in the air, the binding can form between SARS-CoV-2 Spike and fat aerosols, which are entrained in the ventilated airflow and can travel for a longer distance, increasing the chances of airborne transmission of the virus. The enhanced attachment of the virus to equipment surfaces and workers’ clothes makes sanitation challenging and can lead to longer residence time of the virus and impose higher risks to contact transmission. This study helps recommend necessary modifications to sanitation and cleaning procedures in meat processing plants. For example, hosing the floors and workbenches with warm water, heating the surface temporarily before cleaning, and modifying the mode of ventilation to reach a lower humidity can potentially increase the efficiency of removing SARS-CoV-2 from the facilities and providing a safer and cleaner environment to protect workers. Future studies can explore higher S protein concentrations and intermediate temperatures between 0 and 37 °C to further delineate the binding kinetics of the virus proteins. The MD simulation can be performed in the future on S protein and RBD at 0 °C to assess any structural changes in low temperature environments.

## Supplementary Information

Supplementary Information 1.

## Data Availability

All data generated or analyzed during this study are included in this published article [and its Supplementary information files].
